# Removal of Toxic Metals from Sewage Sludge by Acid Hydrolysis Coupled with EDTA Washing in a Closed-Loop Process

**DOI:** 10.3390/ijerph20032544

**Published:** 2023-01-31

**Authors:** Juan Francisco Morales Arteaga, Mojca Zupanc, Matevž Dular, Domen Lestan, Anela Kaurin

**Affiliations:** 1 Agronomy Department, Biotechnical Faculty, University of Ljubljana, Jamnikarjeva 101, 1000 Ljubljana, Slovenia; 2 Faculty of Mechanical Engineering, University of Ljubljana, Askerceva Cesta 6, 1000 Ljubljana, Slovenia; 3 Envit Ltd., Trzaska Cesta 330, 1000 Ljubljana, Slovenia

**Keywords:** sewage sludge, toxic metals, EDTA, hydrolysis, microwaves, cavitation, phosphorus

## Abstract

Contamination with toxic metals prevents the use of sewage sludge (SS) as a soil fertilizer. Hydrodynamic cavitation, thermal microwaving, microwave-assisted alkaline, and acid hydrolysis coupled with ethylenediaminetetraacetate (EDTA) washing were tested as a method to remove toxic metals from SS. Acid hydrolysis coupled with EDTA washing was most effective and was used in a closed-loop process based on ReSoil technology. EDTA and process solutions were recycled at a pH gradient of 12.5–2, which was imposed by the addition of quicklime (CaO) and H_2_SO_4_. An average of 78%-Pb, 76%-Zn, 1%-Cu, and 17%-Cr were removed from SS in five consecutive batches. No wastewater was generated, only solid waste (40%). The EDTA lost in the process (42%) was resupplied in each batch. In a series of batches, the process solutions retained metal removal efficiency and quality. The treatment removed 70% and 23% of P and N, respectively, from SS and increased the leachability of Zn, Cu, Mn, and Fe in the washed SS by 11.7, 6.8, 1.4, and 5.2 times, respectively. Acid hydrolysis coupled with EDTA washing proved to be a technically feasible, closed-loop process but needs further development to reduce reagent, material, and nutrient loss and to reduce toxic emissions from the washed sludge.

## 1. Introduction

In European Union (EU) countries alone, about 90 g of sewage sludge (SS) is produced per person per day, which corresponds to a total of 10 million tons per year [1]. The processing and disposal of SS are one of the most complex environmental issues due to its physical-chemical characteristics: SS contains P, which is a strategic plant nutrient in agriculture, N and C [2], but also toxic metals (TMs), organic pollutants, and pathogenic bacteria, and viruses [3]. TMs such as Zn, Cd, Cr, Cu, Pb, and Ni accumulate in the soil, which limits the direct application of SS to arable land as fertilizer [4]. In some EU countries, e.g., Bulgaria, 50% of SS was used in agriculture [5]. However, in most EU countries, the disposal of untreated SS in the soil is prohibited [6].

The three most common methods for SS treatment are digestion, lime stabilization, and incineration [7]. Anaerobic digestion is usually used in wastewater treatment plants to reduce the volume of activated sludge because of its simple application with low energy requirements, the effective handling of fluctuations, flexibility with temperature and pH changes, and biogas production [8]. Nevertheless, high TMs levels can critically inhibit the anaerobic digestion of SS [9]. The addition of lime effectively mitigates the leaching of TMs through chemical stabilization [10] but does not reduce the total concentration of TMs. The incineration of SS is another common practice. High temperatures oxidize the organic matter, reduce the volume, stabilize the TMs, and make them less harmful. The method requires emission control (e.g., SO_2_ and NO_x_) and can reach high costs due to high energy consumption [11]. Incineration removes organic and biological contaminants but concentrates TMs [12].

Alternative methods for treating SS include the removal of TMs from SS by dissolution with organic acids, e.g., glutamic acid, citric acid, acetic acid, and oxalic acid [13,14,15], and mineral acids, e.g., HNO_3_, HCl, and H_2_SO_4_ [13,16,17,18]. Several studies have focused on the chelation and removal of TMs from SS by ethylenediaminetetraacetate (EDTA) and other strong chelators [19,20,21]. TMs chelation is more selective than acid dissolution and largely preserves the pool of available P in SS [22]. Other methods include thermal treatments such as alkaline hydrolysis, e.g., by Ca(OH)_2_, which efficiently reduces the SS amount, improves SS dewaterability, kills pathogens, and efficiently precipitates TMs and P from the aqueous phase [23,24]. Acid hydrolysis with H_2_SO_4_ is more often applied despite its serious drawbacks, such as the corrosion of equipment, required post-neutralization, and solubilization of TMs and P [25]. Some authors reported a significant reduction in TMs concentration, except for Cu, Hg [25], and Pb [23]. Microwave irradiation imposes a “thermal” effect due to the absorption of microwave energy by water molecules in organic complexes [26] and a “non-thermal effect” by electromagnetic irradiation [27]. This is an energy- and cost-efficient method that reduces waste volume, destroys pathogenic microorganisms, disintegrates SS, and releases extracellular polymeric substances [28,29]. More recently, hydrodynamic cavitation has been introduced as a pretreatment measure to enhance SS anaerobic digestion and aerobic composting [30]. Cavitation is a process of microbubble generation. The lifespan of a bubble is a few microseconds. When the bubble collapses, the energy is released to the surrounding fluid in the form of heat and shock waves [31,32,33]. This method has been shown to reduce the aromaticity of SS and enhance the biodegradation of dissolved organic matter in SS [34].

In our previous study, we showed that washing with EDTA is efficient in removing Pb, Zn, Cu, Cr, Mn, and Fe from SS [35]. The novel closed-loop process, in which chelator and washing/rinsing solutions are treated and recycled in a pH gradient, was introduced. No wastewater, only solid waste, was generated from the process [35]. However, the composition of SS is very complex and depends on the origin and load of the wastewater and the method of wastewater treatment [36,37]. Daily fluctuations in the chemical composition of SS have been reported [38]. Our preliminary experiments indicated that for some SS, the removal of TMs by washing with EDTA alone was not effective enough.

The aim of this study was to couple the novel closed-loop EDTA washing with the SS treatment methods: hydrodynamic cavitation, microwave irradiation, microwave-assisted acid hydrolysis, and microwave-assisted alkaline hydrolysis for more effective removal of TMs. The feasibility of the most efficient method (EDTA washing coupled with SS acid hydrolysis) was tested in several consecutive batches in a closed-loop process. The effect of the treatment on the leachability of residual TMs, and on the concentration/availability of P, N, and K were evaluated.

## 2. Materials and Methods

### 2.1. SS Origin and Chemical Properties

The aerobically treated SS used in this study was obtained from a wastewater treatment plant in Slovenia. For use in the experiments, the SS with a moisture content of more than 80% was air dried at 40 °C until constant weight and ground to <2 mm.

The pH and electrical conductivity (EC) of SS were measured in a suspension with deionized water at a 1/10 (*w*/*V*) ratio [39]. Organic C, total C, and total N were measured after the dry combustion [40,41] using the elemental analyzer (Vario MAX CNS, Elementar Analysensysteme GmbH, Hanau, Germany). The total P was measured spectrophotometrically using the vanadium molybdate yellow method [42]. Plant-available P (as P_2_O_5_) and K (as K_2_O) were measured colorimetrically, and carbonates were determined volumetrically [43].

### 2.2. Hydrodynamic Cavitation Coupled with EDTA Washing of SS

The SS (429 g, dry weight) was suspended in 50 mmol of an L^−1^ Ca-EDTA (Sigma Aldrich, Chemie GmbH, Taufkirchen, Germany ) solution, solid/liquid ration ratio 1/7 (*w*/*V*), with and without the addition of 50 mmol L^−1^ H_2_SO_4_. The SS suspension was cavitated for 5 or 10 min at 10,000 RPM using a rotational generator of hydrodynamic cavitation (RGHC), which acts as a pump and cavitator. RGHC is based on a rotor-stator design in which the rotor and stator have a specially designed geometry that causes periodically repeating pressure oscillations. The concept of RGHC is described in more detail in [44]. After cavitation, the SS in the suspension was further washed in an end-over-end rotator for 50, 55, or 115 min at 20 revolutions per minute (RPM). The washed SS was separated by centrifugation (10 min at 3430× *g*) and rinsed three times with tap water by vortex mixing the suspension in a 110 mL centrifuge tube for 10 s. The same solid/liquid ratio and centrifugation conditions were used for SS washing. The washed/rinsed SS was dried at 105 °C for 24 h, ground in an agate mortar, sieved through a 250 µm sieve, and stored for further analysis.

### 2.3. Microwave Irradiation and EDTA Washing of SS

For microwave irradiation treatment, SS (5 g, dry weight) was suspended in a 50 mmol L^−1^ Ca-EDTA washing solution, solid/liquid ratio 1/7 (*w*/*V*). In some cases, 50 mmol L^−1^ H_2_SO_4_ was added. The SS suspension was first heated at 100 °C for 30 min in a laboratory microwave oven (Mars Xpress, MDS-2000, CM GmbH, Kamp-Lintfort, Germany) and then washed in an end-over-end rotator for 15 min at 20 RPM. The washed SS was rinsed, dried, ground, sieved, and stored for further analysis, as described above.

### 2.4. Microwave-Assisted Acid/Alkaline Hydrolysis and EDTA Washing of SS

For microwave-assisted acid hydrolysis treatments, SS (5 g, dry weight) was suspended in 50 mmol L^−1^ Ca-EDTA washing solution, solid/liquid ratio 1/7 (*w*/*V*), acidified to pH 2 or 3 by the addition of H_2_SO_4_, and heated in a microwave at 100 °C for 30 min or 1 h. Microwave-assisted alkaline hydrolysis was performed in the same way as acid hydrolysis, except for the pH of the SS suspension, which was adjusted to pH 10 by the addition of CaO. The SS in acid/alkaline suspension was further washed in an end-over-end rotator for 15 min at 20 RPM. The washed SS was rinsed, dried, ground, sieved, and stored as described above.

### 2.5. Washing of SS in Closed Process Loop in Series of Batches

**Figure 1:**
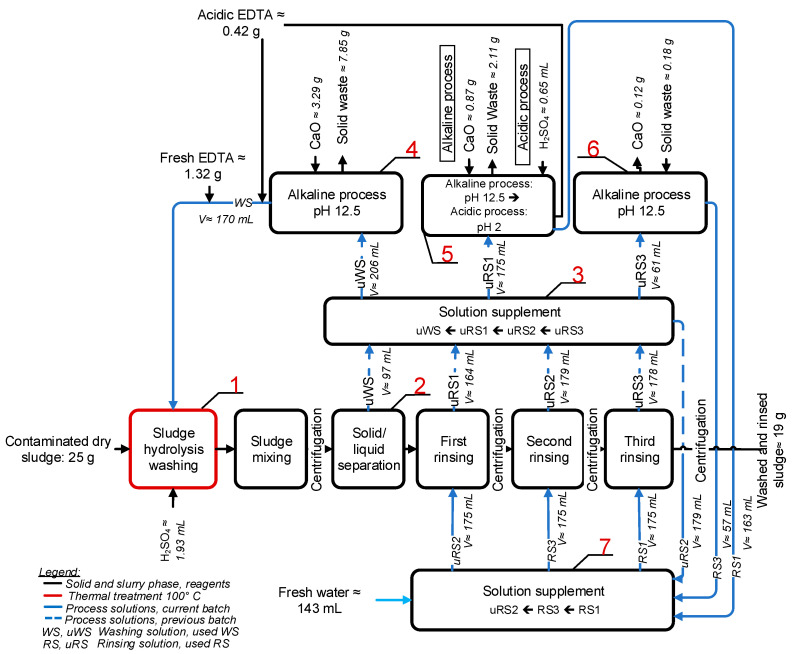
The flowchart of the process with material balance. Process steps: (1) sewage sludge (SS) microwave-assisted acid hydrolysis and EDTA washing, (2) solid/liquid separation and SS rinsing, (3) compensation of water losses, (4) alkalization of uWS, (5) alkalization/acidification of uRS1, (6) alkalization of uRS3, and, finally, (7) the upstream supplement of process solutions with fresh water to reach the final volume. WS, uWS denotes washing and the used washing solution, RS1 and uRS1 represent first rinsing and the used rinsing solution, RS2 and uRS2 represent second rinsing and the used rinsing solution, RS3 and uRS3 represent third rinsing and the used rinsing solution. Blue lines denote the flow of solutions, dashed blue lines denote the flow of solutions from the previous batch, black lines denote the flow of solids.

The SS was acid hydrolyzed and washed with EDTA in a series of 5 consecutive batches in a closed-loop process. The flowchart of the process and the mass balance of the materials used and produced are shown in Figure 1. The dry SS, 25 g per batch, was suspended in a 50 mmol L^−1^ EDTA washing solution (WS) with a solid/liquid ratio of 1/7 (*w*/*V*) in Step 1 (Figure 1), and the suspension was acidified to pH 3 by adding H_2_SO_4_. The suspension was heated in a microwave oven at 100 °C for 1 h for acid hydrolysis. The SS in suspension was then washed with EDTA in an end-over-end rotator at 20 RPM for 15 min. The washed SS was separated from the used WS (uWS) by centrifugation (10 min at 3430× *g*) in Step 2. The SS, which was washed in the first series of batches, was then rinsed three times with tap water (solid/liquid ratio 1/7 (*w*/*V*)) by vortex mixing for 10 s in a 500 mL centrifuge bottle. The suspension was then separated by centrifugation (10 min at 3430× *g*). The SS that was washed in subsequent batches was then rinsed (under the same conditions as described above) with three rinsing solutions (RS) obtained from the previous batch in the following order: used (untreated) second RS (uRS2), treated/recycled third RS (RS3), and treated/recycled first RS (RS1). The used RS (uRS1, uRS2 and uRS3) were obtained as process solutions after the separation of the washed and rinsed SS by centrifugation. uWS, uRS1, and uRS3 were then treated in Steps 4–6 (Figure 1), as explained below, to obtain the recycled WS, RS1, and RS3. uRS2 was not treated and was used as such in the next series of batches. The solid-washed and rinsed SS (washed SS) was the final product of the process.

uWS was supplemented with uRS1 in Step 3 to compensate for water losses due to the hydration of dry SS, microwave heating, and hydration of CaO to Ca(OH)_2_, as explained below. uRS1 was further supplemented with uRS2 and uRS2 with uRS3 to maintain the solid/liquid ratio of 1/7 (*w*/*V*) throughout the series of batches.

uWS, uRS1, and uRS3 were treated and recycled using ReSoil^®^ technology [45] in a pH gradient of 12.5–2. Briefly, uWS was alkalinized to a pH of 12.5 with CaO in Step 4 to precipitate and remove TMs as insoluble hydroxides and excess hydrated lime (Ca(OH)_2_) by centrifugation (10 min at 3430× *g*). EDTA in WS was recycled in the form of Ca-EDTA. uRS1 was alkalinized to a pH of 12.5 in Step 5 to remove TMs and was then acidified to a pH of 2 by H_2_SO_4_ to precipitate and recycle the remaining EDTA in the acidic form (H_4_EDTA). H_4_EDTA was recovered by centrifugation and returned to WS along with fresh Na-EDTA to replenish the EDTA lost during the process, presumably by binding to precipitated Fe hydroxides and to the washed SS. uRS3 was also alkalinized in Step 6 to remove TMs.

The process solutions were compensated for water losses due to the hydration of CaO to Ca(OH)_2_ in Step 7 (Figure 1) in the following order: uRS2 (a process solution that was not treated) was supplemented with RS3 to a total volume of 175 mL, RS3 was supplemented to 175 mL with RS1, and RS1 was supplemented with tap water.

### 2.6. Determination of TMs

To determine the total concentration of TMs in SS, aqua regia extraction was performed using a microwave oven (Mars Xpress, MDS-2000, CM GmbH, Kamp-Lintfort, Germany). The digestate was filtered through a 0.45 µm cellulose acetate membrane and diluted with deionized water [46]. TMs were quantified by flame AAS (Spectr AA-240FS, Varian Optical Spectroscopy Instruments, Hoogeveen, The Netherlands ) and (at low concentration) by graphite cuvette-AAS (GF-AAS 240Z, Agilent Technologies, Santa Clara, CA, USA). The limits of quantification (LOQ) were 10, 10, 30, 20, 20, and 60 µg L^−1^ for Pb, Zn, Cu, Cr, Mn, and Fe, respectively.

### 2.7. Determination of EDTA in Process Solutions

EDTA concentration in the process solutions was determined spectrophotometrically according to Wang et al. [47]. The LOQ was 0.15 mmol L^−1^.

### 2.8. Leachability of Toxic Elements

The concentration of metals in the leachates was measured according to DIN 38414-S4 [39] and the Slovenian national legislation [48]. TMs from SS were extracted with deionized water at a 1/10 ratio after mixing in an end-over-end rotator for 24 h. The method was selected because it proved to be the most repeatable assay for SS [49].

### 2.9. Statistical Analysis

Differences between the original and washed SS were assessed by an analysis of variance (ANOVA). In the case of statically significant interactions, differences between the means of the variables were analyzed using Duncan’s test. All statistical analyses were performed using the R studio program [50].

## 3. Results and Discussion

### 3.1. Chemical Properties of SS

The SS used in this study contained 41, 635, 135, and 36 mg kg^−1^ Pb, Zn, Cu, and Cr, respectively (Table 1). Zn was the main contaminant. The SS also contained 85 and 10,240 mg kg^−1^ Mn and Fe, respectively, which are not considered TMs but readily chelate with EDTA and are removed by washing SS [35]. The TMs concentration in the soil has been shown to increase linearly with increases in the SS application rate [51]. Therefore, the removal of TMs could make SS much more suitable for use as a fertilizer in agriculture.

SS is a complex mixture of macromolecular organic matter, including polysaccharides, proteins, and lipids [52]. TMs are immobilized on these organic materials within the SS flocs or on their surface by biosorption, bioaccumulation, bioprecipitation, and bioreduction [53]. Physical and thermal treatments that aim at releasing TMs from organic matter would likely make TMs more available for chelation with EDTA. Therefore, the washing of SS with EDTA was tested in combination with hydrodynamic cavitation, microwave irradiation, microwave-assisted alkaline hydrolysis, and microwave-assisted acid hydrolysis. The series of simple SS washing experiments (without recycling EDTA and recovering process solutions) was initially set up to select the most efficient combination.

### 3.2. Selection of the Most Efficient Treatment to Remove TMs from SS

In all initial simple SS washing experiments, Ca-EDTA was used in the washing solution because this is the main form of EDTA recycled in the ReSoil^®^ process (Steps 1–7, Figure 1), which was applied next to the in-batch experiments. The addition of H_2_SO_4_ “activated” Ca-EDTA and shortened the time of SS washing by the mechanism described in detail by [45].

Washing with 50 mmol L^−1^ Ca-EDTA and 50 mmol L^−1^ H_2_SO_4_ for 1 h removed 61%-Pb, 24%-Zn, 4%-Cu, 31%-Mn, and 12%-Fe from SS (Treatment No. 1, Table S1). Hanay et al. [54] suggested the use of higher dosages than 0.1 M EDTA in combination with the electrokinetic process to achieve good removal of Cr, Pb, and Zn after EDTA washing. It has been previously shown that hydrodynamic cavitation can release TMs from SS organic matter [34]. The combination of cavitating the SS suspension in 50 mmol L^−1^ Ca-EDTA and 50 mmol H_2_SO_4_ for 5 min followed by washing for 55 min (Treatment No. 2) increased the removal efficiency of Pb, Mn, and Fe by an additional 7%, 15%, and 16%, respectively, compared with Treatment No. 1. Hydrodynamic cavitation did not improve Zn removal. SS washing with 50 mmol L^−1^ Ca-EDTA, when coupled with 5 min of cavitation and extended washing (115 min; Treatment No. 3), increased Zn removal by an additional 23% compared with Treatment No. 2. Increasing the cavitation time to 10 min in Treatment No. 4 (50 mmol L^−1^ EDTA, 50 min washing) did not improve the TMs removal efficiency.

Microwave irradiation (30 min) and SS washing (15 min) with 50 mmol L^−1^ Ca-EDTA (Treatment No. 5) removed 55%-Pb, 27%-Zn, 26%-Mn, and 7%-Fe. The activation of Ca-EDTA by the addition of 50 mmol L^−1^ H_2_SO_4_ enhanced the removal of Pb, Zn, Mn, and Fe to 70%, 43%, 35%, and 30%, respectively (Treatment No. 6). Similar to the previous treatment, Cu and Cr were not removed. Dewil et al. [23] reported that acid hydrolysis alone could reduce the total content of Cd, Cr, and Zn from SS but did not remove Cu and Pb. For microwave-assisted alkaline hydrolysis, the pH of the SS suspension in 50 mmol L^−1^ Ca-EDTA washing solution was adjusted to pH 10 by adding quicklime, CaO, and heating for 30 min, and then washing for 15 min (Treatment No. 7). The treatment removed 10% of the Mn and had no effect on the TMs. The reason for this inefficiency is probably the saturation of the washing solution with Ca ions, which form strong complexes with EDTA under alkaline conditions [55] and, in this way, interfere with the chelation of other metal cations.

Microwave-assisted acid hydrolysis (30 min, pH 2 adjusted with H_2_SO_4_) and SS washing (15 min) with 50 mmol L^−1^ EDTA removed 85%-Pb, 66%-Zn, 55%-Mn, and 64%-Fe (Treatment No. 8). The treatment was efficient because acid hydrolysis releases TMs in mobile forms [56], which presumably chelate readily with EDTA. The extended time of acid hydrolysis (1 h at pH 3) further increased the TMs removal efficiency to 91%-Pb, 73%-Zn, 3%-Cu, 63%-Mn, and 73%-Fe (Treatment No. 9). Acid hydrolysis (1 h) at pH 2 (Treatment No. 10) marginally increased the removal of Pb, Zn, Mn, and Fe by the additional 6%, 1%, 3%, and 2%, respectively, compared to Treatment No. 9.

Microwave-assisted acid hydrolysis coupled with the EDTA washing applied in Treatment No. 9 was therefore selected for further SS washing with recycling EDTA and process solutions in a closed-loop process in a series of five consecutive batches.

The array of simple SS washing tests showed little or no removal of Cu and Cr (Table S1). Both elements are known to form strong bonds with organic matter [34,57]. For example, Dewil et al. [23] reported that acid hydrolysis was unable to release Cu by breaking Cu-organic matter complexes.

### 3.3. Acid Hydrolysis and EDTA Washing of SS in a Closed-Loop Process

The suspension of SS in 50 mmol L^−1^ EDTA (*w*/*V* ratio 1/7) was acidified to pH 3 with H_2_SO_4_ acid hydrolyzed at 100 °C for 1 h and washed for 15 min. The flowchart of the batch process is depicted in Figure 1. For the process solutions, the used washing and rinsing solutions (uWS, uRS) were treated in a pH gradient with CaO and H_2_SO_4_ (ReSoil^®^), resulting in recycled washing and rinsing solutions (WS, RS). The background chemical mechanisms of the ReSoil^®^ process, including the alkaline substitution of TMs in the EDTA chelate by Ca, alkaline co-precipitation of TMs and Fe, EDTA recovery as Ca-EDTA, acid precipitation and EDTA recovery of H_4_EDTA, the removal of excess reagents as insoluble CaSO_4_, and Ca-EDTA activation by H_2_SO_4_, were explained in detail by [45].

SS was treated in a series of five consecutive batches in a closed loop; no wastewater was generated, and 0.4 g of solid waste per g of treated SS was produced. Removal efficiencies ranged from 75–81%-Pb, 67–80%-Zn, 0–6%-Cu, 12–24%-Cr, 61–70%-Mn, and 66–76%-Fe (Table 2). According to the EU Directive 86/278/EEC [58] and Slovenian legislation [59], the untreated, original SS met the requirements as a soil fertilizer. However, TMs are persistent and accumulate in the environment over time; thus, reducing their content is crucial, as explained in Section 3.1.

**Figure 2:**
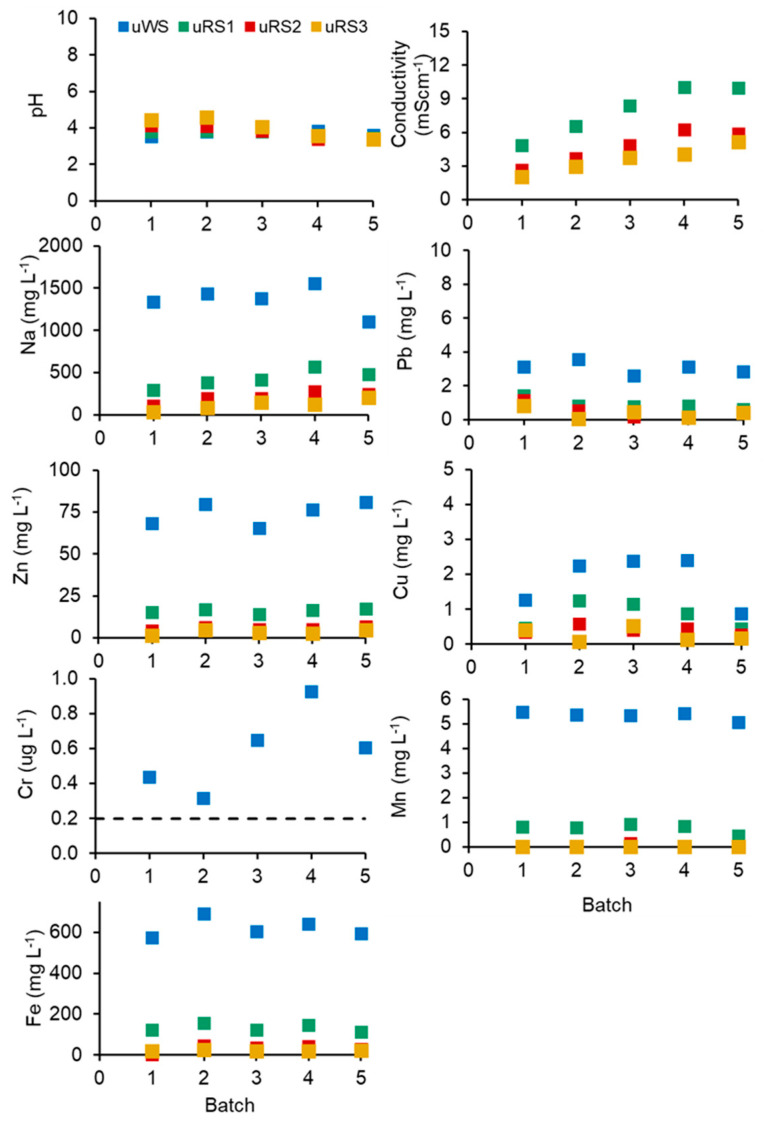
The properties of used washing, first, second, and third rinsing solutions (uWS, uRS1, uRS2, and uRS3, respectively) over the five successive washing batches.

**Figure 3:**
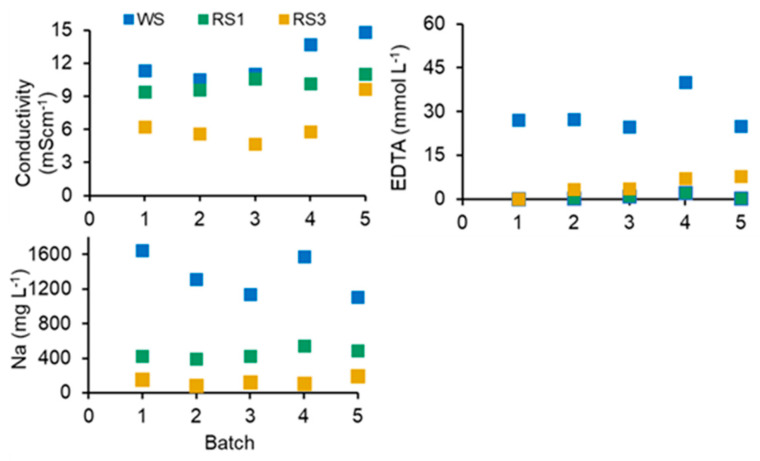
The properties of the recycled washing, first, and third rinse solutions (WS, RS1, and RS3, respectively) over the five successive washing batches.

The removal of Zn as the main SS contaminant increased slightly in the closed-loop process by an additional 3% on average, while the removal of Pb decreased by an additional 13% on average compared to Treatment No. 9 (Table S1). The Cu removal remained very limited. The Cu removal efficiency was 0% for all batches except batch four. The possible reason for Cu removal in the fourth batch is the inhomogeneity of the sludge and the high variability of toxic metal speciation [38]. The SS in batch four probably contained a small fraction of Cu in an EDTA-available form; thus, removal was very low (Table 2). However, the closed-loop process removed some Cr (Table 2), which was not the case with the simple SS washing test (Table S1). The presumed reason for the differences in the removal of TMs is the high concentration of Na^+^ in WS and uWS and in RS and uRS (Figure 2 and Figure 3).

Na ions could interfere with the desorption of TMs from the substrates [60], causing the de-aggregation of SS flocks [61] and the plasmolysis of microbial cells within flocks [62]. In preliminary simple experiments (Table S1), deionized water was used for SS washing and rinsing.

The consistent properties and quality of process solutions are critical for the efficiency and sustainability of a closed-loop process. No increasing trends were observed in the concentrations of TMs and other measured elements in the used washing and rinsing solutions (uWS, uRS1, uRS2, uRS3) (Figure 2). The increase in conductivity from batches one to three reached a plateau in batches four and five. The concentration of Pb, Zn, Cu, Cr, Mn, and Fe in the recycled WS, RS1, and RS3 (Figure 3) was below the limit of quantification (LOQ), proving the efficiency of the ReSoil^®^ process. The concentration of EDTA and Na, as well as the conductivity of the process solutions fluctuated in a series of batches (Figure 3). These fluctuations were presumably related to slight differences in the efficiency of EDTA recycling in the ReSoil^®^ process from batch to batch and to EDTA losses due to binding to organic matter in the washed SS. Consequently, EDTA had to be re-supplied with an average of 21 mmol L^−1^ fresh Na-EDTA in WS (Figure 1). The fluctuations in conductivity, presumably followed the fluctuations of Na in the process solutions but did not lead to a permanent upward trend (Figure 3).

SS was rinsed three times (Figure 1) to remove the TMs mobilized from SS solid phases by acid hydrolysis and EDTA chelation. Efficient rinsing was ensured by a very low (<LOQ) concentration of TMs in recycled RS (Figure 3) compared with much higher concentrations of TMs in uRS (Figure 2). Nevertheless, a statistically significant increase in the leachability of Zn, Cu, Mn, and Fe from washed SS by 11.7, 6.8, 1.4, and 5.2 times, respectively, was measured compared to the original SS using the DIN 38414-S4 [39] test (Table 3). The leachability of Pb and Cr from the washed SS was still well below the concentration limits considered hazardous by 111 and 36 times, respectively. The leachability of Zn and Cu exceeded the hazardous limit by 1.4 times for both TMs. The excess was not large, and an additional SS rinsing cycle might have been sufficient to mitigate Zn and Cr leachability.

Another possible approach to reduce the leachability of TMs could be the addition of zero-valent iron (ZVI) to the washed SS prior to solid/liquid separation (Step 2 in Figure 1). Hydrated ZVI quickly forms an oxide-hydroxide shell around the ZVI core, which strongly adsorbs TMs [63]. For example, [64] has shown that the addition of ZVI effectively curbs the emission of TMs from EDTA-washed soil.

Since metals are often present in microbial cells or are adsorbed by extracellular polymeric substances (EPS) [65], it has been shown that microwave irradiation treatment can destroy the cell structures and EPS of sludge [66], leading to the release of TMs in the liquid phase, and, probably, EDTA was able to bind TMs to its molecule more easily in our experiments than in the treatment without microwave irradiation. Another reason for the high TMs removal is the high temperature applied to SS. It has been reported that at high temperatures, the diffusivity of ions increases, resulting in a higher TMs extraction rate [67]. The third reason for the high TMs removal by the addition of H_2_SO_4_ is that TMs are often bound to organic complex molecules, which are destroyed during the hydrolysis process and released in the form of soluble salts (at the prevailing pH) [25].

Regarding Cu, our results were comparable to Neyens et al. [25], who reported that Cu was not released after the acid hydrolysis of SS [25]. The plausible reason for the lower Cu removal is that it forms strong metal–organic chelate bonds, and the greater the organic bond strength, the less soluble the complex. Therefore, Cu is one of the least soluble metal-organic chelate complexes [68].

### 3.4. Depletion of Nutrients from Washed SS

The removal of TMs allows for the safer use of SS for soil nutrition. However, acid hydrolysis coupled with EDTA washing (Figure 1) removed two-thirds of the P from SS and reduced P phytoavailability 13.7 times (Table 1). In addition, the treatment reduced the total N by 1.3 times and the concentration of plant-available K (assessed as K_2_O) by 12.8 times. The hydrolysis of SS with mineral and organic acids is known to cause the dissolution of P and other nutrients from SS [69]. In comparison, SS hydrodynamic cavitation for 5 min followed by SS washing for 115 min with 50 mmol L^−1^ EDTA (Treatment No. 3, Table S1) resulted in a loss of only 9% (20,585 ± 125.51 mg kg^−1^ total P in Treatment No. 3) of the total P but was less efficient in removing TMs.

The total C and total organic C content after SS acid hydrolysis coupled with EDTA washing were less affected, and both decreased by only 9–11%. H_2_SO_4_ and EDTA also dissolved a small number of carbonates and an additional 1.57% from SS (Table 1). The overall depletion of N, P, and K was the major drawback of the washing process.

## 4. Conclusions

We demonstrated the technical feasibility of acid hydrolysis coupled with EDTA washing for the efficient removal of TMs from SS. Chelator and process solutions were recycled by the ReSoil^®^ process. Wastewater was not generated, and the recycled process solutions retained their properties and quality throughout the series of five batches. The increased concentration of TMs in water extracts of washed SS was measured, and the washed SS lost most of P, N, and K. These issues will be addressed in our future research: TMs absorbents will be tested to curb emissions, other SS pretreatment methods and, specifically, hydrodynamic cavitation, will be tried before SS washing to reduce the loss of nutrients. Larger pilot-scale experiments will be required to address the cost efficiency of the novel SS treatment process.

## Figures and Tables

**Table 1:** Metal concentrations and properties of original and washed sewage sludge (SS) after acid hydrolysis coupled with EDTA. Data for metal concentrations in original SS are given as the average ± standard error of three subsamples taken from the homogenized bulk, and for washed SS as the calculated average from batches 1–5. Properties of original SS refer to the homogenized bulk sample, and washed SS to the combined sample from batches 1–5.

Metals (mg kg^−1^)	Original SS	Washed SS
Pb	40.6 ± 0.2	9 ± 0.4
Zn	634.9 ± 0.8	156 ± 14.4
Cu	134.6 ± 1.0	141 ± 4.4
Cr	36.0 ± 1.4	28 ± 1.4
Mn	84.6 ± 0.1	29 ± 0.8
Fe	10,240 ± 239	2803 ± 169
Properties		
pH	6.91	4.35
EC (mS cm^−1^)	6.67	2.92
Total P (%)	22,680 ± 145	6798 ± 111
P_2_O_5_ (mg 100 g^−1^)	2065 ± 7	150 ± 17
Total N (%)	6.5 ± 0.0	5.0 ± 0.0
Total organic C (%)	42.4 ± 0.1	38.3 ± 0.0
Total C (%)	42.7 ± 0.1	38.4 ± 0.0
K_2_O (mg 100 g^−1^)	877.4 ± 4.1	68.6 ± 5.6
CaCO_3_ (%)	2.50 ± 0.12	0.93 ± 0.07

Limits of toxic metals (TMs) for SS to make it suitable to be used as fertilizer according to the EU Directive 86/278/EEC and Slovenian legislation Ur. l. RS, št. 10/14. Uredba o odlagališčih odpadkov: 250, 1200, 300, and 200 mg kg^−1^ Pb, Zn, Cu, and Cr, respectively (dry weight).

**Table 2:** The removal efficiency of metals from washed sewage sludge (SS) in a series of five consecutive batches.

Batch Number	Removal Efficiency (%)					
	Pb	Zn	Cu	Cr	Mn	Fe
1	81	67	0	14	61	66
2	78	77	0	24	68	74
3	76	76	0	13	67	73
4	75	79	6	20	69	75
5	79	80	0	12	70	76

**Table 3:** Leaching of metals from original and washed sewage sludge (SS). Data are given as average ± standard error of three subsamples taken from the homogenized bulk of original SS and from a combined sample of washed SS from batches one–five. Different letters indicate significant differences between treatments according to Duncan’s test (*p* < 0.05).

Metals (mg kg^−1^)	Original SS	Washed SS	DIN 38414-S4 *
Pb	0.08 ± 0.02 ^a^	0.09 ± 0.01 ^a^	10
Zn	5.99 ± 0.34 ^a^	70.16 ± 1.37 ^b^	50
Cu	10.19 ± 0.26 ^a^	69.09 ± 2.84 ^b^	50
Cr	0.18 ± 0.02 ^a^	0.28 ± 0.04 ^a^	10
Mn	1.36 ± 0.05 ^a^	1.86 ± 0.05 ^b^	/
Fe	47.77 ± 9.33 ^a^	249.68 ± 16.73 ^b^	/

* Concentrations stipulated as hazardous (DIN 38414-S4, Council Decision 2003/33/EC).

## Data Availability

All materials and datasets can be accessed on request from the corresponding author.

## Supplementary Materials

The following supporting information can be downloaded at: https://www.mdpi.com/article/10.3390/ijerph20032544/s1, Table S1: Preliminary laboratory-scale sewage sludge (SS) washing experiments. Optimization of washing solution (WS) for efficient removal of Pb, Zn, Cu, Cr, Mn, and Fe from contaminated SS. The percentages for the removal of the elements are given.
