# Supplementary material for: Removal of Toxic Metals from Sewage Sludge by Acid Hydrolysis Coupled with EDTA Washing in a Closed-Loop Process
Source: Int J Environ Res Public Health. 2023 Jan 31;20(3):2544. doi: 10.3390/ijerph20032544 (PMC9916202; doi:10.3390/ijerph20032544)
Supplement: Supplementary file 1 [file ijerph-20-02544-s001.zip › ijerph-2141765-supplementary.pdf]

International Journal of
*Environmental Research and Public Health*

MDPI

**Supplementary Materials:**

**Table S1.** Preliminary laboratory-scale sewage sludge (SS) washing experiments. Optimization of washing solution (WS) for efficient removal of Pb, Zn, Cu, Cr, Mn, and Fe from contaminated SS. The percentages for the removal of the elements are given.

| Treatment No. | Treatment Description | Toxic Element Removal (%) | | | | | |
|---|---|---|---|---|---|---|---|
| | | Pb | Zn | Cu | Cr | Mn | Fe |
| | EDTA washing | | | | | | |
| 1 | A 1 h extraction with 50 mmol $L^{-1}$ EDTA and 50 mmol $L^{-1}$ $H_2SO_4$. The ratio used in the experiment was 1:7. The SS was mixed with the EDTA solution and $H_2SO_4$. | 61 | 24 | 4 | 0 | 31 | 12 |
| | Cavitation experiments | | | | | | |
| 2 | A 1 h extraction with 50 mmol $L^{-1}$ EDTA and 50 mmol $L^{-1}$ $H_2SO_4$. The ratio used in the experiment was 1:7. The suspension (SS/washing solution) was first cavitated for 5 min at 10,000 RPM and then mixed for 55 min. | 68 | 25 | 0 | 0 | 46 | 28 |
| 3 | A 2 h total extraction time with 50 mmol $L^{-1}$ EDTA. The ratio used in the experiment was 1:7. The suspension (SS/washing solution) was first cavitated for 5 min at 10,000 RPM and then mixed for 115 min. | 57 | 48 | 2 | 0 | 35 | 10 |
| 4 | A 1 h total extraction time with 50 mmol $L^{-1}$ EDTA. The ratio used in the experiment was 1:7. The suspension (SS/washing solution) was first cavitated for 10 min at 10,000 RPM and then mixed for 50 min. | 42 | 14 | 0 | 0 | 0 | 0 |
| | Microwave-assisted experiments | | | | | | |
| 5 | A 45 min total extraction time with 50 mmol $L^{-1}$ EDTA. The ratio used in the experiment was 1:7. The suspension (SS/washing solution) was first microwave heated for 30 min at 100 °C and then mixed for 15 min in a rotatory mixer at 20 RPM. | 55 | 27 | 0 | 0 | 26 | 7 |
| 6 | A 45 min total extraction time with 50 mmol $L^{-1}$ EDTA and 50 mmol $L^{-1}$ $H_2SO_4$. The ratio used in the experiment was 1:7. The suspension (SS/washing solution) was first microwave heated for 30 min at 100 °C and then mixed for 15 min in a rotatory mixer at 20 RPM. | 70 | 43 | 0 | 0 | 35 | 30 |
| 7 | A 45 min total extraction time with 50 mmol $L^{-1}$ EDTA pH 10. pH was adjusted by the addition of CaO. The ratio used in the experiment was 1:7. The suspension (SS/washing solution) was first microwave heated for 30 min at 100 °C and then mixed for 15 min in a rotatory mixer at 20 RPM. | 1 | 1 | 0 | 0 | 10 | 0 |
| 8 | A 45 min total extraction time with 50 mmol $L^{-1}$ EDTA pH 3. pH was adjusted by the addition of $H_2SO_4$. The ratio used in the experiment was 1:7. The suspension (SS/washing solution) was first microwave heated for 30 min at 100 °C and then mixed for 15 min in a rotatory mixer at 20 RPM. | 85 | 66 | 0 | 0 | 55 | 64 |
| 9 | A 75 min total extraction time with 50 mmol $L^{-1}$ EDTA pH 3. pH was adjusted by the addition of $H_2SO_4$. The ratio used in the experiment was 1:7. The suspension (SS/washing solution) was first microwave heated for 1 h at | 91 | 73 | 3 | 0 | 63 | 73 |

| | | | | | | | |
|---|---|---|---|---|---|---|---|
| | 100 °C and then mixed for 15 min in a rotatory mixer at 20 RPM. | | | | | | |
| 10 | A 75 min total extraction time with 50 mmol $L^{-1}$ EDTA pH 2. pH was adjusted by the addition of $H_2SO_4$. The ratio used in the experiment was 1:7. The suspension (SS/washing solution) was first microwave heated for 1 h at 100 °C and then mixed for 15 min in a rotatory mixer at 20 RPM. | 97 | 74 | 0 | 0 | 66 | 75 |
| 11 | A 75 min total extraction time with fresh water. The ratio used in the experiment was 1:7. The suspension (SS/washing solution) was first microwave heated for 1 h at 100 °C and then mixed for 15 min in a rotatory mixer at 20 RPM. | 0 | 0 | 0 | 0 | 0 | 0 |
